# Short Bowel Patients Treated for Two Years with Glucagon-Like Peptide 2: Effects on Intestinal Morphology and Absorption, Renal Function, Bone and Body Composition, and Muscle Function

**DOI:** 10.1155/2009/616054

**Published:** 2009-08-20

**Authors:** P. B. Jeppesen, P. Lund, I. B. Gottschalck, H. B. Nielsen, J. J. Holst, J. Mortensen, S. S. Poulsen, B. Quistorff, P. B. Mortensen

**Affiliations:** ^1^Department of Gastroenterology CA-2121, Rigshospitalet, Blegdamsvej 9, DK-2100 Copenhagen, Denmark; ^2^Department of Medicine CA-2121, Rigshospitalet, Blegdamsvej 9, DK-2100 Copenhagen, Denmark; ^3^Department of Anesthesia, Rigshospitalet, Blegdamsvej 9, DK-2100 Copenhagen, Denmark; ^4^Department of Biomedical Sciences, The Panum Institute, Nørre Alle 20, DK-2200 Copenhagen, Denmark; ^5^Department of Clinical Physiology and Nuclear Medicin, Rigshospitalet, Blegdamsvej 9, DK-2100 Copenhagen, Denmark

## Abstract

*Background and aims*. In a short-term study, Glucagon-like peptide 2 (GLP-2) has been shown to improve intestinal absorption in short bowel syndrome (SBS) patients. This study describes longitudinal changes in relation to GLP-2 treatment for two years. *Methods*. GLP-2, 400 micrograms, s.c.,TID, were offered, to eleven SBS patients keeping parenteral support constant. 72-hour nutritional balance studies were performed at baseline, weeks 13, 26, 52 during two years intermitted by an 8-week washout period. In addition, mucosal morphometrics, renal function (by creatinine clearance), body composition and bone mineral density (by DEXA), biochemical markers of bone turnover (by s-CTX and osteocalcin, PTH and vitamin D), and muscle function (NMR, lungfunction, exercise test) were measured. *Results*. GLP-2 compliance was >93%. Three of eleven patients did not complete the study. In the remaining 8 patients, GLP-2 significantly reduced the fecal wet weight from approximately 3.0 to approximately 2.0 kg/day. This was accompanied by a decline in the oral wet weight intake, maintaining intestinal wet weight absorption and urinary weight constant. Renal function improved. No significant changes were demonstrated in energy intake or absorption, and GLP-2 did not significantly affect mucosal morphology, body composition, bone mineral density or muscle function. *Conclusions*. GLP-2 treatment reduces fecal weight by approximately 1000 g/d and enables SBS patients to maintain their intestinal fluid and electrolyte absorption at lower oral intakes. This was accompanied by a 28% improvement in creatinine clearance.

## 1. Introduction

GLP-2 is cosecreted with GLP-1 from the enteroendocrine L cells following nutrient ingestion [1]. In animal and human studies, GLP-2 decreases gastric acid secretion [2], inhibits antral gastric emptying [3, 4] and upregulates intestinal blood-flow [5]. In addition, GLP-2 has been demonstrated to have trophic effects on the intestinal mucosa [6–9] and positive effects on the absorptive function [10–12]. Furthermore, GLP-2 decreases bone resorption [13]. 

 Theoretically, treatment with GLP-2 could reduce the rapid gastric emptying and hypersecretion and increase the intestinal absorption in short bowel syndrome (SBS) patients. In addition, GLP-2 could decelerate bone losses and diminish osteoporosis often described in these patients [14]. Therefore, native GLP-2 and a dipeptidyl-peptidase IV (DPP-IV) degradation-resistant gly-2 GLP-2 analog [7], Teduglutide, have been evaluated in short-term, “proof of concept” studies in the treatment of SBS patients [15, 16]. The positive effects on intestinal morphology were confirmed by increases in small intestinal villus heights and crypt depths, and the positive absorptive effects by increases in the wet weight, and to a minor degree, in the energy absorption. Positive effects on bone mineral content were also described [17]. However, the long-term effects of GLP-2 treatment in SBS patients remain to be evaluated. 

 This open-label study describes the effects of GLP-2, 400 mcg offered for subcutaneous injection TID, to eleven patients for two consecutive years intermitted by an 8-week washout period. As a part of the study design, parenteral support was kept constant during the two years in order to evaluate longitudinal changes in the intestinal absorption and dietary intake in relation to GLP-2 treatment. Mucosal morphology, renal function, body composition, bone mineral density and muscle function were also recorded.

## 2. Material and Methods

### 2.1. Patients

Eleven SBS patients (3 female, 8 male; 47 ± 11 years; remnant small bowel 157 ± 66 cm; 2 with a colon; 7 had intestinal failure, 3 receiving parenteral fluids and electrolytes exclusively and 4 receiving parenteral nutrition; 4 had intestinal insufficiency and did not need parenteral nutrition or fluid) were recruited to the study based on a fecal energy excretion exceeding 2.0 MJ/d (measured at a previous admission) or a remnant small bowel of 200 cm or less (measured perioperatively from the ligament of Treitz) (Table 1). One patient had previously received native GLP-2 (OBJ) and two teduglutide (LM and HRM) in the short-term experiments, whereas the remaining 8 patients were GLP-2 treatment naïve. All patients, except FL and LM, took antidiarrheal medication (codein, loperamide or opium) and 6 of eleven patients (HM, OB, EFP, JE, JHJ and UDJ) took antisecretory agents (omeprazole).

### 2.2. Study Protocol

Over the two years, the patients were admitted at least eight times to the hospital for 72-hours evaluations (Table 2). After the baseline evaluations, the patients were given native GLP-2, 400 mcg TID, subcutaneous, for one year. For these studies we employed synthetic human GLP-2, as described previously [15]. During the first year, the patients were scheduled for readmissions at 13, 26 and 52 weeks (abbreviated Y1-W13, Y1-W26, Y1-W52, resp.,). After the first readmission at week 13, the patients were given an option to test a double dose of GLP-2, 800 mcg TID for 3 weeks. The patients, who accepted, were readmitted for an extra 72-hour nutrient balance study at week 17 (Y1-W17). After completing this evaluation, the original GLP-2 dose was reintroduced. GLP-2 treatment was discontinued for 8 weeks after the first 52 weeks of treatment. After a 72-hour washout evaluation, the GLP-2 treatment, 400 mcg TID, was reintroduced and evaluations were repeated during admission at 13, 26 and 52 weeks during the second year of treatment (abbreviated Y2-W13, Y2-W26, Y2-W52, resp.,). In relation to the week 26 readmission, during the second year of GLP-2 treatment, the patients were given cholylsarcosine bile acid replacement therapy, 2 grams TID, two days prior to the admission and during the 72-hour balance studies [18, 19]. Cholylsarcosine was supplied as the water soluble sodium salt, >99% pure by HPLC and thin-layer chromatography, and was packed into gelatine capsules (250 mg/capsule) [20]. Cholylsarcosine was taken in relation to the three main meals and in conjunction with subcutaneous GLP-2 injections.

#### 2.2.1. Morphological Analysis

At least two small bowel biopsy specimens were obtained before GLP-2 treatment at baseline and repeated at week 52, year 1, in 6 of 7 patients with a jejunostomy. Histologic sections of the biopsies were analyzed by morphometric methods (Image pro plus) as described previously [15].

#### 2.2.2. Fluid, Electrolyte and Nutrient Balance Studies

The study—and collection—period began at 9 o’clock on the first day of admission, where patients were requested to urinate, defecate or empty their stoma-bags. During the 72-hour balance periods, all ad libitum oral intakeand stomal output were weighed, and the contents of energy (bomb calorimetry), carbohydrate (Englyst's method), nitrogen (Kjeldahl's method), fat (gas liquid chromatography), sodium and potassium (flame photometry), calcium and magnesium (atomic absorption spectrophotometry) were determined as previously described [21, 22]. The absolute intestinal absorption was calculated as the difference between the ingested and excreted and the relative as the absolute absorption divided by oral intake. 

 The medication and parenteral supplements were fixed according to the status at baseline.

#### 2.2.3. Urine Creatinine Excretion and Creatinine Clearence

Urinary creatinine was measured at 505 nm as a pikrat-creatinine complex using a standard hospital analytical technique according to the method of Jaffe and the 72-hour output calculated. The creatinine clearance was calculated by diving daily urinary creatine excretion by the plasma creatinine concentration.

#### 2.2.4. Assessment of Body Weight, Body Composition and Bone Mineral Density

The fasting body weights were measured every morning after emptying of the bladder and stoma-bags, before breakfast, using a leveled platform scale, and were calculated as the mean for 4 consecutive days. Body composition (BC) and bone mineral density (BMD) of the posterior-anterior spine, hip and total body were measured by Dual-energy X-ray Absorptiometry (Norland XR-36 DXA densitometer, Norland Corp., Fort Atkinson, WI., USA).

#### 2.2.5. Biochemical Markers of Bone Turnover

Bone resorption was assessed from the concentration of s-CTX (Serum CrossLabs one step ELISA; Nordic Bioscience, Denmark) [23]. Bone formation was assessed from the concentration of s-osteocalcin (Osteocalcin N-MID ELISA assay, Nordic Bioscience, Denmark) [23].

#### 2.2.6. Evaluation of Lung Function and Maximal Inspiratory and Expiratory Force

Lung function was tested by dynamic and static spirometry and measurement of single-breath diffusion capacity of carbon monoxide (MasterLab plethysmograph, Jaeger, Germany) according to the recommendations of the European Respiratory Society [24, 25]. Results were expressed in absolute values and as percentage of predicted values calculated according to European reference equations. Respiratory muscle strength was assessed by measurement of maximal expiratory and inspiratory pressures [26].

#### 2.2.7. Evaluation of Maximal Aerobic ATP Turnover of Skeletal Muscle Mitochondria

The ^31^P NMR spectroscopy investigations were conducted in a short 26 cm bore magnet at 2.9 T as described previously [27–29]. The examination of the forearm flexor muscles and the tibialis anterior muscle of the lower leg was done as two separate experiments on the same day and both protocols involved: a 5 minutes rest, three minutes of dynamic exercise at 50% of maximal voluntary contraction followed by 10 minutes of recovery. The aim of the measurements was to obtain from the pH and PCr recovery a measure of the capacity of aerobic ATP synthesis. 

 On a separate day, patients performed an exercise test on a modified Krogh cycling ergometer with the upper body in a 45° position [30]. Exercise was maintained at 60 round per minute with an increase in workload by 50 Watt every third minute until exhaustion. Breath-by-breath measurements of pulmonary O_2_ consumption (VO_2_) were made with an online gas analyzer (CPX/D, Medical Graphics, St. Paul, MN) and data were averaged every 30 seconds. Heart rate was recorded noninvasively [31].

### 2.3. Ethics

The Ethics Committee for Medical Research in Copenhagen, Denmark (KF 01-235/98) approved the protocol. Procedures followed were in accordance with the ethical standards of the Helsinki Declaration of 1975, as revised in 1983. Patients signed informed consent before entrance to the study.

### 2.4. Statistics

As the vast majority of data was normally distributed, the results are presented as means ± standard deviations. The differences between admissions periods were tested with a Friedman repeated measures analysis of variance on ranks on using the SigmaStat for Windows Version 2.0 (1992–1995, Jandel Corporation, Erkrath, Germany) in patients completing the study. For comparisons of admission periods to the baseline period, the Dunnett method was used as the post hoc test. A value of *P* < .05 was considered significant.

## 3. Results

Results regarding compliance, safety, adverse events, quality of life and treatment satisfaction are presented separately [32]. In summary, all patients injected more than 93% of the prescribed GLP-2. Three of eleven patients did not complete the study. Two of these patients experienced abdominal pain, and in one patient, the investigator discontinued GLP-2 treatment, since the feedback from the patient regarding the administration of GLP-2 was lacking [32]. Abdominal pain could be a consequence of providing an intestinotrophic agent to patients with a relative stenosis, and caution should probably be taken, when prescribing GLP-2 or analogs to patients with a relative intestinal stenosis, a narrow stoma, or a history of chronic abdominal pain [32]. 

### 3.1. Morphological Analysis

Villus height increased in 4 out of 6 patients, but overall no significant changes were seen (316 ± 53 *μ*m versus 346 ± 87 *μ*m, *P* = .37) in relation to GLP-2 treatment. Crypt depth increased in 3 patients, and decreased in 3, but overall no change was seen (160 ± 63 *μ*m versus 140 ± 24 *μ*m, *P* = .44). 

#### 3.1.1. Fluid, Electrolyte and Nutrient Balance Studies (Table 3)

Fecal wet weight was significantly reduced by approximately one liter/d at all time intervals after the initiation of GLP-2 treatment (range 629 ± 862 g/d to 1201 ± 1389 g/d). The average reduction the first year was 811 g/d and not different from the average reduction the second year of 1081 g/d, and no trend of a further increase in the effect on fecal weight beyond the 13th week of treatment was seen (Table 3). The fecal wet weight excretion reverted to baseline levels in the washout period after one year of treatment, but as for the first year the effect on fecal weight was fully regained 13 weeks after treatment was reinstituted the second year. The reduction in fecal wet weight was also seen in the two SBS patients with colon-in-continuity and numerically equaled the findings in the patients with a jejunostomy. 

 The reduction in fecal wet weight excretion was accompanied by a reduction in oral intake. However, in contrast to the prompt effect on fecal weight, the effect on oral intake gradually increased over the first year by an average from 449 ± 1061 g/d after 13 weeks of treatment to 611 ± 813 g/d after 26 weeks to finally 913 ± 996 g/d after 52 weeks (Y1-W52). Also in contrast to fecal excretions, oral intake was not reversed to baseline values during the eight weeks where treatment was stopped, but was only halved to an intermediate level of 453 ± 641 g/d. However, in the second year the effect on oral intake was already back to levels of 902 ± 919 g/d after 13 weeks, comparable to effects it took 52 weeks of treatment to reach the first year. In the second year, no further effect was seen on oral intake beyond week 13, and the effect leveled off at a decreased oral intake of 775 ± 886 g/d and 1058 ± 1082 g/d after 26 and 52 weeks of treatment, respectively. The decrease in fecal output resulted in a nearly comparable decrease in oral intake rendering absolute effect on intestinal absorption unaltered although intestinal absorption in percentage of oral intake increased (Table 3). 

 Similarly, the numerical increase in urine volume was small and did not reach statistical significance (on average 291 g/d the first year and 238 g/d the second year) rendering the overall urine excretion rather constant throughout the GLP-2 treatment periods in spite of the reduced oral intake. Fecal sodium excretions were reduced at all admissions (on average 53 mmol/day the first year and 58 mmol/day the second year) in relation to GLP-2 treatment and reverted to baseline levels during the washout period. Similarly, urinary sodium excretions increased at most admissions (54 mmol/day the first year and 24 mmol/day the second year). Although the decrease in fecal excretion and the increase in urinary excretion indicate that intestinal sodium absorption must have been increased, the *P*-value for this was not significant (*P*-value of .06 from the data in Table 3). Treatment did not affect oral sodium intake in contrast to oral wet weight intake. 

 Both dietary intake and fecal excretions of potassium decreased with no significant change in absorption although urinary excretions increased during several sessions of measurements. Consistent changes in dietary intake, fecal excretion and intestinal absorption of calcium and magnesium were not seen. Urinary excretion of calcium did not increase in relation to GLP-2 treatment (*P* = .08), whereas urinary magnesium excretion increased significantly (*P* = .001) from a baseline of 2 ± 2 mmol/d to values ranging from 3 ± 3 mmol/d to 5 ± 3 mmol/d. 

 The results on the energy and macronutrient balances are presented in Table 4. On an average, treatment with GLP-2 numerically decreased oral energy intake by 349 kJ/d in the first year and 1182 kJ/d in the second year, which was less than 3% and 8% of baseline intake and not significant (*P* = .69, Table 4). Fecal excretion of energy numerically decreased on an average by 448 kJ/d in the first year and 1438 kJ/d in the second year. None of these changes were significant. However, the combination of GLP-2 and cholylsarcosine appeared successful in reducing fecal excretions by a total of 1852 kJ/d (~31%), which partly appeared to be caused by a cholylsarcosine associated 42% decrease in fecal fat excretion of 28 g/d (1094 kJ/d, *P* = .005), as GLP-2 alone did not influence fecal fat (Table 4). Nevertheless, the accompanying reduction in dietary intake reduced the overall gain in energy absorption to a negligible amount of less than 100 kJ/d except for the period where cholylsarcosine was added, which resulted in a numerical increase in absorption of 701 kJ/day or 8% of baseline, but still not enough to reach significance. 

 Fecal excretions of carbohydrate and protein (nitrogen) did not decrease (*P* = .08) with no demonstrable increase in intestinal absorption (Table 4).

Six of the eight patients completing the study opted to try the double dose of GLP-2, 800 mcg TID for 3 weeks and were readmitted for an extra 72-hour nutrient balance study (Y1-W17, Patients HRM, LM, GL, JE, JHJ and JV). Compared to effects at week 13, doubling the GLP-2 dose for three weeks did not significantly affect wet weight intake (4761 ± 1780 g/d versus 4561 ± 1730 g/d, *P* = .84), fecal wet weight excretion (2469 ± 1468 g/d versus 2484 ± 1678 g/d, *P* = 1.00) or urine wet weight excretion (1634 ± 857 g/d versus 1752 ± 848 g/d, *P* = .18). Sodium, potassium, calcium and magnesium balances were unaffected by the doubling of the GLP-2 dose. Likewise, compared to effects at week 13, doubling the GLP-2 dose for three weeks did not significantly affect diet energy intake (16123 ± 5373 kJ/d versus 16178 ± 6354, *P* = .56), fecal energy excretion (6646 ± 3817 kJ/d versus 6657 ± 4745 kJ/d, *P* = 1.00) or carbohydrate, fat or protein balances.

#### 3.1.2. Urine Creatinine Excretion and Creatinine Clearance (Table 5)

On an average GLP-2 increased creatinine clearance by 20 mL/min or 28% in both years of treatment (*P* = .04) from a baseline value of 73 ± 22 mL/min (normal range 48–150 mL/min) to a range between 91 ± 14 mL/min and 97 ± 21 mL/min during the first year and a range between 85 ± 36 mL/min and 105 ± 23 mL/min during the second year. Creatinine clearence reverted to baseline levels (67 ± 43 mL/min) during the washout period. Corresponding changes were seen in plasma and urine creatinine.

### 3.2. Assessment of Body Weight and Composition, Bone Mineral Density, Biochemical Markers of Bone Turnover (S-Crosslabs and S-Osteocalcin), Vitamin D and PTH (Table 6)

Although the body weight numerically increased from 69.2 ± 9.1 at baseline to 72.6 ± 13.7 after two years of GLP-2 treatment, this did not reach statistical significance (*P* = .25). No significant changes occurred regarding lean body mass and fat mass, and bone mineral content was constant in relation to the two years of GLP-2 treatment. 

 Significant, but sporadic, differences between admission periods were demonstrated regarding bone mineral density of the femoral neck and the distal radius and ulna, but the post hoc analyses were nonsignificant. No significant changes were seen regarding z-scores of the femoral neck or the spine. S-crosslabs decreased significantly at week 13. Vitamin D fluctuations were probably related to the season of the year. Osteocalcin and PTH remained constant throughout the two years of GLP-2 treatment.

### 3.3. Evaluation of Physical Strength and Mitochondrial Function of Skeletal Muscle (Lung Function and Maximal Inspiratory and Expiratory Force, NMR Turnover Test and Exercise Test)

Measurements of lung function at baseline showed that 7 of 8 (88%) patients had an obstructive ventilatory defect. Five of 8 (63%) patients had a restrictive ventilatory defect and 8 of 8 (100%) patients had decreased diffusion capacity. No change was observed in relation to the GLP-2 treatment in flow indices as for example, FEV1, static volumes as total lung capacity, residual volume and vital capacity or diffusion capacity for carbon monoxide. Yet, functional residual capacity decreased significantly from 144 ± 23% at baseline to 128 ± 28% (*P* < .001) at week 52. Respiratory muscle strength was within the normal limits and did not change in relation to the GLP-2 treatment. 

 The intension of the ^31^P NMR spectroscopic experiments was to test, if the capacity of aerobic ATP synthesis of the skeletal muscle was changed by the GLP-2 treatment. The measurements were performed in two muscle groups, the tibialis anterior, which is primarily a red muscle (type 1) [29] and the forearm flexor muscle, which is primarily of type 2 [33]. Table 7 shows the results at baseline before treatment was initiated. The differences observed between the two muscles are similar to what has been observed previously [29]. The data from the subsequent measurements at week 13 is not shown, since no significant changes were observed in any of the parameters recorded. 

 In relation to the exercise test, the resting heart rate and VO_2_ were 78 ± 4 bpm and 0.3 ± 0.05 L/min, at baseline, respectively, and when patients were exhausted during cycling the heart rate and VO_2_ increased to 161 ± 5 bpm and 1.8 ± 0.9 L/min, respectively, (*P* < .05). Thirteen weeks of treatment with GLP-2 did not change resting and exercise heart rate and VO_2_.

## 4. Discussion

In contrast to the previous short-term studies, where active efforts were made to enforce strict compliance to injections and adhesion to fixed diets in hospital-like settings, this study aimed at describing the consequences of introducing and expanding long-term GLP-2 treatment to the daily life of the short bowel patients. However, parenteral support was deliberately maintained constant. GLP-2 was well tolerated as demonstrated by the high compliance in the SBS patients completing this study (≥94%) but also in the patients discontinuing GLP-2 treatment (≥93%). 

 The effect of long-term GLP-2 treatment on intestinal morphology was evaluated in biopsies taken at baseline and after a year of GLP-2 treatment in six patients with a jejunostomy. No significant changes in the villus height (10 ± 25%, *P* = .37) or the crypt depth (−5 ± 28%, *P* = .44) were observed. This was in accordance with the findings in the initial 35 days GLP-2 study [15], where 400 *μ*g of native GLP-2 was given subcutaneously twice daily (corresponding to 0.013 ± 0.002 mg/kg/d, a range of 0.011–0.017 mg/kg/d). No changes were seen in villus height (10 ± 19%, *P* = .14) or the crypt depth (18 ± 42%, *P* = 0.28). In contrast, 21 days of teduglutide in doses ranging from 0.03 to 0.10 mg/kg/d [16] increased villus heights by 38 ± 45% (*P* = .030) and crypt depth increased 22 ± 18% (*P* = .010) in eight short bowel jejunostomy patients. The more pronounced positive effects of teduglutide may be due to the prolonged half-life of the peptide and subsequently by the larger area under the curve, or simply be related to the larger doses used in that study. The finding of continuously elevated endogenous GLP-2 levels and increased mucosal growth in short bowel patients with colon-in continuity and in obese patients subjected to jejunoileal bypass operations supports the “area under the curve” theory. In these patients, the endogenous meal-stimulated peak-concentrations of GLP-2 are much lower than the pharmacologically induced peaks, but a continuous endogenous GLP-2 secretion occurs, even after a night of fasting. Thus, the increases in villus height and crypt depths in short bowel patients with a jejunostomy treated with either native GLP-2 or teduglutide is still significantly less than the 80% increases in villus height demonstrated in patients with jejunoileal bypass operations [34] and the 2–300% increases in villus heights described in patients with enteroglucagonomas [35, 36]. However, it is important to notice that local concentrations of GLP-2 delivered by paracrine secretions may not reflect plasma levels. 

 The magnitude of the effects of GLP-2 and teduglutide on bowel morphology is important in the discussion of the potential for cancerous growth attributable to these peptides. GLP-2, and to a greater degree, teduglutide, have been shown to promote an increase in the growth of mucosal neoplasms in mice developing colonic tumors in response to the methylating carcinogen 1,2-dimethylhydralazine [37]. Despite the fact that the small bowel is the longest region of the alimentary tract, neoplasms of the small bowel are very uncommon. Therefore, in our practice, screening for small bowel neoplasms is not considered prior to initiation of GLP-2 treatment or during treatment. On the other hand, cancer of the colon and rectum is the fourth most common newly diagnosed cancer in The United States and Europe. The colorectal adenomas that may predispose to this condition are even more frequent. It could be argued, that screening prior to the initiation of GLP-2 treatment would be appropriate in short bowel patients with a preserved colon. However, as stated, the patients, who have undergone jejono-ileal bypass operations, have continuously elevated endogenous GLP-2 plasma concentrations. In these patients, the changes in the functional remnant jejunum clearly supersede changes obtained by pharmacological intervention, and the colonic crypts are also more enlarged. Thus, it is suggested, that there is no indication for regular colonoscopic surveillance. Although it will probably take another 10–20 years to establish whether jejuno-ileal bypass is associated with an increased risk of large bowel cancer in humans, the findings, so far, are negative [38]. The colon was normal before and after GLP-2 treatment in the two SBS patients with colon-in continuity in this study. 

 The effects on changes in intestinal absorption in relation to GLP-2 treatment were evaluated in balance studies. Significant, clinically relevant and reproducible effects could be demonstrated on intestinal wet weight excretion and absorption over the two years of GLP-2 treatment. GLP-2, 400 mcg/d TID (corresponding to 0.018 ± 0.002 mg/kg/d, range 0.014–0.020 mg/d), reduced the fecal wet weight excretion from baseline values of approximately 3.0 to approximately 2.0 kg/d after the first year of GLP-2 treatment a reduction of the same magnitude was demonstrated during the second year. This reduction in the fecal wet weight excretion exceed the effects described in the initial 35 days GLP-2 study (400 mcg BID corresponding to 0.013 ± 0.002 mg/kg/d, range 0.011–0.017 mg/kg/d), where the fecal wet weight decreased from 3.2 kg/d to 2.8 kg/d, and in the 21 days teduglutide study (doses ranging from 0.03 to 0.15 mg/kg/d OD), where fecal wet weight decreased from 2.8 to 2.1 kg/d. However, in these studies the oral intakes were maintained constant as a part of the protocol. 

 In this study, the fecal wet weight excretion was not reduced by doubling the GLP-2 dose in six patients in this study for 3 weeks. Thus, the maximal effect may have been reached by the TID injection regimen. 

 The decrease in fecal wet weight excretion was gradually accompanied by a decline in the oral wet weight intake, thereby maintaining the intestinal wet weight absorption and urinary weight constant. In this study, parenteral support was kept constant during the entire study unless otherwise indicated by the patient and the investigator, and it was deliberately kept constant during the balance periods. An alternative approach would have been to taper the parenteral support according to the urinary production keeping the hyperphagic intake constant. 

 Since, the oral intake did not increase, and since the parenteral supplement of fluid and electrolytes was kept constant, the significant increases in urinary sodium, potassium and magnesium and the borderline significant increase in the urinary calcium excretion reflect the beneficial effects of GLP-2 on the intestinal absorption of these electrolytes. Although urinary volume did not increase significantly in relation to GLP-2 treatment, the renal function, evaluated by the renal creatinine clearance, improved significantly in this study. Renal impairment is a documented complication in HPN patients and is at least partially due to a relative chronic dehydration [39]. Since parenteral support is frequently administered during nighttime, many short bowel patients experience relative over-hydration during nighttime and relative dehydration during daytime. We speculate, that GLP-2 treatment reduces the amplitude of the daily fluctuations in the fluid-balance of the short bowel patients, thereby reducing the daily interval, when dehydration is present. The positive effects on renal function are also reflected in the decrease in plasma-creatinine and the increase in plasma-CO_2_-total in relation to GLP-2 treatment. 

 Although, the fecal energy excretion tended to decrease in relation to long-term GLP-2 treatment, this did not affect the overall intestinal energy absorption, since the dietary energy intake tended to decrease accordingly. In the short-term studies employing native GLP-2 and teduglutide, the dietary intakes were fixed, and this may explain the positive trend towards an increase in the overall energy absorption. In the short-term study with native GLP-2, the absolute energy absorption tended to increase by approximately 400 ± 600 kJ/d (~100 kcal/d; *P* = .09). In the study using the dipeptidyl peptidase IV-resistant GLP-2 analog teduglutide, in doses 0.03 to 0.15 mg/kg/d, in 16 short-bowel patients (six with remnant parts of the colon), fecal energy excretion was reduced by approximately 800 kJ/d (*P* = .04), but this only translated into a significant increase in intestinal absorption of approximately 1000 kJ/d in a post hoc defined subset of patients with high dietary compliance during balance studies. In none of these three studies significant changes in the absorption of individual macronutrients were demonstrated. Although, a type-2 error is likely, the combination of GLP-2 and cholylsarcosine only tended to increase the fat absorption (36 ± 35 g/d versus 59 ± 26 g/d, *P* = .08). However, all the patients receiving 2 grams of cholylsarcisine TID (8 capsules TID) responded, that they would not be able to continue this treatment long-term, and the clinical applicability therefore seems limited. 

 Originally, we believed that the marginal effects on intestinal energy absorption in the initial 35-days native GLP-2 study could explain the rather dramatic changes seen in body weight and body composition [15]. In the light of the negative findings on intestinal energy absorption, body weight and body composition in this long-term GLP-2 study, the increases described in body weight, lean body mass and the concurrent reduction in fat mass in the initial native GLP-2 study could simply reflect a transient fluid retention in the initial phase of GLP-2 treatment prior to an adjustment to the new equilibrium. 

 In contrast to the initial native GLP-2 study, no changes were observed regarding bone mineral content in relation to long-term GLP-2 treatment. Again, acute fluctuations in calcium absorption or changes in bone formation and resorption could account for these conflicting findings. The GLP-2 dose currently suggested in the treatment of osteoporosis is 1600 *μ*g given at bedtime. Thus, the lower dose and the timing of injections may also explain the lack of effect of GLP-2 on bone mineral density in this study. In the initial study by Haderslev et al., positive effects on urinary excretions of markers of bone resorption, deoxypyridinoline and pyridioline, were described [17]. However, to compensate for day-to-day variation in urinary volume, these data were corrected for urinary creatinine excretion on the assumption that for each patient the 24-hour urinary creatinine excretion was constant. However, since GLP-2 seems to affect urinary creatinine excretion by other means, the interpretation of these results may be incorrect. Thus, the clinical role of GLP-2 in the treatment of osteoporosis remains uncertain. It is currently investigated in postmenopausal women in a long-term, double blind, placebocontrolled trial, and studies on the physiological basis of the suggested effect of GLP-2 on bone resorption and formation are ongoing. 

 Since GLP-2 was initially believed to improve nutritional status in short bowel patients, we included measures of physical function in this study, including ^31^P NMRS testing maximal aerobic ATP turn-over of red and white skeletal muscle. In accordance with the negative findings on body composition, none of these measures improved in relation to long-term GLP-2 treatment. 

 In conclusion, the main effect of GLP-2 treatment in patients with short bowel syndrome, where the parenteral support is kept constant, is a reduction in fecal losses of fluid and electrolytes. The effects on energy and macronutrient absorption are minor. This enables the patients to maintain their intestinal fluid and electrolyte absorption at lower oral intakes. A reduction in the amplitude in the fluctuations of the daily fluid-balance in relation to GLP-2 treatment may explain the beneficial effects on renal function. 

 An alternative approach to benefit from the positive effects of GLP-2 on intestinal absorption is to wean the SBS patients from parenteral support by encouraging them to maintain a hyperphagic oral intake during GLP-2 treatment. If the need for parenteral fluid support could be reduced by the magnitude of the reduction in fecal wet weight excretions, that is, approximately 1000 g/d, theoretically approximately 10–15% of short bowel patients with intestinal failure (based on evaluations of the HPN volumes needed in the Danish HPN-cohort) would be able to regain intestinal autonomy, and be weaned from HPN. They could have their central line removed in relation to treatment with GLP-2. It must, however, be emphasized, that all current data is obtained in open, nonrandomized studies and should be regarded as preliminary. However, the weaning strategy is currently tested in double blind, placebo-controlled trial. Although the preliminary results are inconsistent, it is likely that GLP-2 or analogs eventually could contribute to the limited treatments armamentarium of the short bowel syndrome.

## Figures and Tables

**Table 1:** Patient characteristics at baseline.

Patient ID	Gender/age (years)/diagnosis	Body mass index (kg/m^2^)	Small bowel (cm)	Colon-in cont. (%)	Time since last surgery (years)	Wet weight intake (kg/d)	Fecal wet weight excretion (kg/d)	Parenteral fluid (L/d)	Diet energy intake (MJ/d)	Fecal energy excretion (MJ/d)	Patenteral energy (MJ/d)	Duration of HPN (years)	Time on GLP-2(days, 365 + 365 ~2 years)
HRM	F/47/CD	21.3	150	0	2.5	5.2	3.7	1.0	13.5	7.1	0.8	2.5	365 + 365
LM	M/27/CD	21.0	70	75	2.5	3.9	2.3	2.3	11.8	6.0	6.4	2.5	365 + 365
OB	M/49/CD	25.5	150	0	11.6	3.1	1.3	0.5	13.2	4.6	0.0	11.6	232 + 0, Abd. pain
GL	M/53/CD	17.3	180	0	20.6	3.2	1.6	no	16.8	4.4	no	no	365 + 365
JP	M/24/CD	20.4	130	100	2.3	3.1	0.7	1.8	16.4	4.6	4.5	2.3	365 + 160, Abd. pain
EFP	F/55/CD	17.5	50	0	0.5	2.2	4.9	3.1	8.0	7.1	5.5	1.7	365 + 174, unrel. feedback
JE	M/44/UC compl.	27.2	200	0	1.6	5.9	3.9	no	13.8	5.5	no	no	365 + 365
JHJ	M/55/UC compl.	22.2	200	0	2.3	4.6	1.9	1.3	17.8	2.8	0.4	2.3	365 + 365
UDJ	F/50/UC compl.	25.8	150	0	0.9	2.8	2.2	2.9	8.8	2.2	2.7	2.9	365 + 365
JV	M/67/CD	22.6	180	0	12.1	8.7	7.1	no	28.2	18.1	no	no	365 + 365
FVL	M/59/CD	20.1	290	0	5.8	3.1	1.2	no	7.8	1.3	no	no	365 + 365

M: male, F: female, CD: Crohns disease, UC compl.: Ulcerative colitis complications, Abd. Pain: Abdominal pain, Unrel. Feedback: Unreliable feedback.

**Table 2:** Schedule of procedures and evaluations.

	1st year				2nd year			
	Baseline	Week 13	Week 26	Week 52	Washout (8 weeks post-treatment)	Week 13	Week 26	Week 52
Treatment	None	GLP-2	GLP-2	GLP-2	None	GLP-2	GLP-2 + Cholyl-sarcosine*	GLP-2
Intestinal biopsy (morphometry)	×			×				
72-h balance on diet, feces, urine (wet weight, na+, k+, mg2+, ca2+, energy, fat, carbohydrate, protein)	×	×	×	×	×	×	×	×
72-h Urinary creatinine excretion	×	×	×	×	×	×	×	×
DEXA (body weight + body composition + bone mineral content)	×	×	×	×	×	×	×	×
Vitamin D, PTH	×	×	×	×	×	×	×	×
Bone resorption and formation markers(s-crosslabs, osteocalcin)	×	×	×	×	×			
NMR scan	×	×						
lung function + in- and expiratory force	×	×	×	×				
exercise test on bike	×	×						

*Cholylsarcosine was given two days prior to the admission and during the 72-hour balance studies.

**Table 3:** Diet, feces, absorption (diet-feces), and urine excretion of wet weight, sodium, potassium, calcium, and magnesium.

(mean ± SD, *n* = 8)	1st year				2nd year				
	Baseline	Week 13	Week 26	Week 52	Washout	Week 13	Week 26	Week 52	*P*-value*
Treatment	None	GLP-2	GLP-2	GLP-2	None	GLP-2	GLP-2 + Cholyl-sarcosine#	GLP-2	

Wet weight (g/d)									
* *Diet	4680 ± 1945	4232 ± 1813 §	4069 ± 1523 §	3768 ± 1355 §	4228 ± 1589 §	3779 ± 1254 §	3905 ± 1293 §	3623 ± 1047 §	.03
* *Feces	2995 ± 1905	2136 ± 1388 §	2366 ± 391 §	2048 ± 1158 §	2759 ± 1371	1853 ± 1093 §	2094 ± 1139 §	1794 ± 771 §	<.001
* *Abs. absorption	1686 ± 578	2096 ± 600	1702 ± 784	1720 ± 506	1468 ± 627	1926 ± 854	1811 ± 679	1929 ± 656	.11
* *Rel. absorption	39 ± 16	52 ± 13 §	41 ± 16	48 ± 14	35 ± 17	52 ± 18 §	47 ± 18	50 ± 15	<.001
* *Urine	1407 ± 860	1740 ± 756	1720 ± 749	1635 ± 774	1410 ± 807	1855 ± 1002	1528 ± 917	1551 ± 596	.16
Sodium (mmol/d)									
* *Diet	238 ± 116	233 ± 126	228 ± 117	209 ± 118	223 ± 109	222 ± 113	203 ± 101	216 ± 104	.41
* *Feces	264 ± 132	197 ± 113 §	234 ± 96 §	203 ± 96 §	250 ± 150	198 ± 108 §	226 ± 119 §	195 ± 79 §	.001
* *Abs. absorption	−26 ± 84	36 ± 64	−6 ± 89	6 ± 71	−27 ± 126	25 ± 74	−23 ± 96	21 ± 61	.06
* *Rel. absorption	−24 ± 72	13 ± 29	−25 ± 81	−18 ± 74	−27 ± 78	−8 ± 92	−32 ± 95	−2 ± 55	.15
* *Urine	77 ± 139	139 ± 80 §	133 ± 50 §	122 ± 55 §	59 ± 72	124 ± 64 §	78 ± 61	100 ± 66	.001
Potassium (mmol/d)									
* *Diet	117 ± 45	107 ± 43 §	111 ± 38 §	100 ± 43 §	104 ± 43 §	93 ± 34 §	90 ± 26 §	90 ± 24 §	.007
* *Feces	49 ± 41	32 ± 31 §	31 ± 28 §	28 ± 24 §	46 ± 30	24 ± 21 §	26 ± 23 §	23 ± 22 §	<.001
* *Abs. absorption	68 ± 43	76 ± 37	80 ± 28	73 ± 36	58 ± 38	70 ± 36	64 ± 30	67 ± 26	.21
* *Rel. absorption	58 ± 24	72 ± 28	74 ± 19 §	72 ± 24	56 ± 30	73 ± 28	72 ± 25	75 ± 22 §	<.001
* *Urine	62 ± 16	73 ± 13	86 ± 25 §	73 ± 19	49 ± 16	87 ± 19 §	64 ± 24	86 ± 20 §	<.001
Calcium (mmol/d)									
* *Diet	49 ± 30	44 ± 27	42 ± 20	34 ± 20 §	39 ± 28 §	32 ± 19 §	31 ± 18 §	32 ± 20 §	<.001
* *Feces	66 ± 52	65 ± 42	70 ± 41	61 ± 42	63 ± 43	50 ± 31 §	49 ± 32 §	45 ± 24 §	<.001
* *Abs. absorption	−17 ± 35	−21 ± 26	−28 ± 27 §	−27 ± 28 §	−25 ± 25 §	−18 ± 20	−18 ± 26	−13 ± 20	.009
* *Rel. absorption	−39 ± 59	−55 ± 60	−72 ± 63	−89 ± 82	−87 ± 76	−69 ± 60	−77 ± 112	−66 ± 85	.24
* *Urine	3 ± 3	3 ± 3	4 ± 4	4 ± 4	4 ± 4	5 ± 4	5 ± 4	4 ± 3	.08
Magnesium (mmol/d)									
* *Diet	19 ± 8	18 ± 8	19 ± 7	17 ± 7 §	18 ± 7	16 ± 6 §	16 ± 5 §	15 ± 4 §	.03
* *Feces	32 ± 27	31 ± 32	34 ± 33	33 ± 35	34 ± 30	29 ± 27	28 ± 25	30 ± 29	.01
* *Abs. absorption	−12 ± 21	−12 ± 25	−15 ± 27	−16 ± 28	−16 ± 25	−13 ± 23	−12 ± 22	−15 ± 26	.10
* *Rel. absorption	−51 ± 80	−42 ± 89	−60 ± 92	−67 ± 99	−73 ± 105	−61 ± 108	−60 ± 119	−79 ± 128	.17
* *Urine	2 ± 2	3 ± 3 §	4 ± 3 §	4 ± 4 §	3 ± 3 §	5 ± 3 §	4 ± 2 §	4 ± 2 §	.001

*Friedman repeated measures analysis of variance on ranks in patients completing the study. For comparisons of admission periods to the baseline period, the Dunnett method was used as the post hoc test. § ~ *P* < .05. #Cholylsarcosine was given two days prior to the admission and during the 72-hour balance studies.

**Table 4:** Diet, feces, and absorption (diet-feces) of energy, fat, carbohydrate, and protein.

(mean ± SD, *n* = 8)	1st year				2nd year				
	Baseline	Week 13	Week 26	Week 52	Washout	Week 13	Week 26	Week 52	*P*-value*
Treatment	None	GLP-2	GLP-2	GLP-2	None	GLP-2	GLP-2 + Cholyl-sarcosine#	GLP-2	

Energy (kJ/d)									
* *Diet	14810 ± 6429	14421 ± 5556	14958 ± 5485	14001 ± 5445	15277 ± 5926	13729 ± 5369	13658 ± 4821	13494 ± 4025	.69
* *Feces	5928 ± 5305	5400 ± 3968	5799 ± 4095	5240 ± 3668	6332 ± 4197	4838 ± 3469	4075 ± 2866	4555 ± 2596	.02
* *Abs. absorption	8882 ± 3339	9021 ± 2500	9159 ± 2151	8761 ± 2461	8945 ± 2655	8892 ± 2786	9583 ± 2649	8940 ± 2419	.82
* *Rel. absorption	64 ± 18	66 ± 16	64 ± 13	65 ± 12	61 ± 16	66 ± 15	72 ± 12	67 ± 14	.03
Fat (g/d)									
* *Diet	103 ± 43	113 ± 44	114 ± 46	103 ± 40	109 ± 46	104 ± 44	98 ± 45	103 ± 42	.22
* *Feces	67 ± 62	69 ± 61	70 ± 58	61 ± 58	78 ± 63	58 ± 56	39 ± 40 §	54 ± 47	.005
* *Abs. absorption	36 ± 35	44 ± 25	44 ± 20	42 ± 33	31 ± 29	46 ± 28	59 ± 26	48 ± 24	.11
* *Rel. absorption	43 ± 34	48 ± 36	46 ± 29	50 ± 37	35 ± 36	50 ± 33	64 ± 27 §	50 ± 34	.002
Carbohydrate (g/d)									
* *Diet	374 ± 145	345 ± 128	354 ± 129	341 ± 126	382 ± 140	342 ± 129	355 ± 107	369 ± 102	.62
* *Feces	66 ± 60	57 ± 43	59 ± 40	57 ± 37	70 ± 42	52 ± 33	50 ± 31	51 ± 19	.08
* *Abs. absorption	308 ± 109	288 ± 97	295 ± 100	284 ± 96	311 ± 106	290 ± 107	306 ± 81	318 ± 90	.53
* *Rel. absorption	84 ± 9	84 ± 6	84 ± 5	84 ± 5	83 ± 6	85 ± 5	87 ± 4	86 ± 3	.18
Protein (g/d)									
* *Diet	105 ± 51	101 ± 48	107 ± 44	96 ± 52	115 ± 54	99 ± 41	95 ± 39	102 ± 41	.15
* *Feces	50 ± 35	46 ± 31	47 ± 27	46 ± 29	57 ± 35	43 ± 34	42 ± 22	43 ± 22	.08
* *Abs. absorption	55 ± 36	54 ± 31	60 ± 25	49 ± 33	58 ± 33	56 ± 29	53 ± 33	59 ± 32	.76
* *Rel. absorption	52 ± 22	55 ± 19	57 ± 13	50 ± 18	51 ± 21	55 ± 19	54 ± 18	56 ± 18	.50

*Friedman repeated measures analysis of variance on ranks in patients completing the study. For comparisons of admission periods to the baseline period, the Dunnett method was used as the post hoc test. § ~ *P* < .05. #Cholylsarcosine was given two days prior to the admission and during the 72-hour balance studies.

**Table 5:** Plasma creatinine, urine creatinine, and creatinine clearence.

(mean ± SD, *n* = 8)	1st year				2nd year				
	Baseline	Week 13	Week 26	Week 52	Washout	Week 13	Week 26	Week 52	*P*-value*
Treatment	None	GLP-2	GLP-2	GLP-2	None	GLP-2	GLP-2 + Cholyl-sarcosine#	GLP-2	
Plasma Creatinine (mmol/l)	0.103 ± 0.019	0.097 ± 0.019 §	0.096 ± 0.019 §	0.095 ± 0.020 §	0.094 ± 0.023 §	0.088 ± 0.018 §	0.101 ± 0.029 §	0.086 ± 0.024 §	.01
Urine Creatinine (mmol/d)	11 ± 3	13 ± 4	13 ± 4	13 ± 4	8 ± 4	11 ± 3	12 ± 5	13 ± 4	.016
Creatinine Clearence (mL/min)	73 ± 22	91 ± 14 §	97 ± 21 §	93 ± 28 §	67 ± 43	90 ± 26 §	85 ± 36 §	105 ± 23 §	.04

*Friedman repeated measures analysis of variance on ranks in patients completing the study. For comparisons of admission periods to the baseline period, the Dunnett method was used as the post hoc test. § ~ P<.05. #Cholylsarcosine was given two days prior to the admission and during the 72-hour balance studies.

**Table 6:** Body weight and composition, bone mineral density, biochemical markers of bone turnover (s-crosslabs and s-osteocalcin), vitamin D and PTH.

(mean ± SD)	1st year				2nd year				
	Baseline	Week 13	Week 26	Week 52	Washout	Week 13	Week 26	Week 52	*P*-value*
Treatment	None	GLP-2	GLP-2	GLP-2	None	GLP-2	GLP-2 + Cholyl-sarcosine#	GLP-2	

Body composition, kg									
* *Body weight	69.2 ± 9.1	70.8 ± 9.5	71.2 ± 9.7	70.2 ± 12.3	70.2 ± 14.0	71.8 ± 12.9	71.3 ± 12.3	72.6 ± 13.7	.25
* *Lean body mass	45.7 ± 9.5	47.3 ± 9.3	47.4 ± 9.9	48.0 ± 10.0	46.7 ± 9.6	48.0 ± 10.0	47.1 ± 9.9	46.9 ± 10.3	.46
* *Fat mass	20.1 ± 8.9	19.4 ± 8.8	20.2 ± 9.0	19.3 ± 8.8	20.0 ± 8.2	20.7 ± 8.9	20.7 ± 8.6	21.8 ± 8.7	.07
* *Bone mineral content	2.67 ± 0.27	2.71 ± 0.32	2.71 ± 0.29	2.73 ± 0.30	2.70 ± 0.29	2.74 ± 0.29	2.74 ± 0.29	2.73 ± 0.26	.75
Bone mineral density, g/cm^2^									
* *Spine	0.97 ± 0.25	0.96 ± 0.26	0.97 ± 0.26	0.95 ± 0.24	0.95 ± 0.24	0.95 ± 0.22	0.96 ± 0.23	0.96 ± 0.22	.49
* *Femoral neck	0.79 ± 0.14	0.80 ± 0.16	0.79 ± 0.14	0.78 ± 0.14	0.78 ± 0.13	0.76 ± 0.13	0.78 ± 0.12	0.77 ± 0.14	.04
* *Dist. Rad+Uln	0.37 ± 0.05	0.37 ± 0.05	0.36 ± 0.05	0.37 ± 0.05	0.37 ± 0.05	0.37 ± 0.05	0.38 ± 0.05	0.37 ± 0.05	.02
* *Prox. Rad+Uln	0.77 ± 0.06	0.77 ± 0.07	0.77 ± 0.06	0.78 ± 0.06	0.78 ± 0.07	0.78 ± 0.06	0.78 ± 0.06	0.78 ± 0.07	.27
* *Prox. Rad	0.77 ± 0.07	0.77 ± 0.06	0.76 ± 0.06	0.77 ± 0.05	0.77 ± 0.06	0.77 ± 0.06	0.78 ± 0.06	0.78 ± 0.07	.06
Spine, Z-Score	−0.70 ± 1.78	−0.80 ± 1.86	−0.71 ± 1.89	−0.57 ± 1.83	−0.82 ± 1.81	−0.82 ± 1.67	−0.83 ± 1.70	−0.71 ± 1.68	.37
Fem. Neck, Z-Score	−0.85 ± 1.10	−0.73 ± 1.19	−0.84 ± 1.04	−0.86 ± 1.04	−0.85 ± 1.11	−1.00 ± 0.96	−0.85 ± 0.94	−0.94 ± 1.07	.23
S-Crosslabs, ng/mL	0.44 ± 0.19	0.25 ± 0.11 §	0.32 ± 0.14	0.40 ± 0.24	0.49 ± 0.32	%	%	%	.04
S-Osteocalcin, ng/L	24 ± 12	24 ± 10	21 ± 9	25 ± 15	24 ± 16	%	%	%	.75
P-PTH, pmol/L	4.5 ± 3.2	4.7 ± 2.8	4.3 ± 2.0	5.0 ± 2.4	3.8 ± 1.7	4.4 ± 2.3	5.1 ± 3.4	4.9 ± 2.2	.92
P-Vitamin-D, nmol/L	79 ± 28	57 ± 25	55 ± 31	91 ± 49	78 ± 37	87 ± 52	89 ± 63	92 ± 44	.02

*Friedman repeated measures analysis of variance on ranks in patients completing the study. For comparisons of admission periods to the baseline period, the Dunnett method was used as the post hoc test. § ~ *P* < .05. #Cholylsarcosine was given two days prior to the admission and during the 72-hour balance studies.

**Table 7:** ^31^PMRS measurements on primarily red (tibialis anterior) or white (forearm) skeletal muscle of subjects before treatment with GLP2. Tibialis anterior represents a primarily “red” and the forearm flexor group a primarily “white” muscle. The first 4 lines are recorded at rest where PCr is given as mM. The subsequent 4 lines are recorded after a 1 min of maximal contraction. The PCr and pH change is the difference between the resting value and the value measured by the end of exercise. The T_0.5_ values are half times for recovery (s), assuming monoexponential recovery after exercise (27).

	Forearm Flexors	Tibialis Anterior
*At rest:*		
PCr	24.3 ± 1.7	23.5 ± 2.3
PCr/ATP	4.2 ± 0.5	4.3 ± 0.4
PCr/Pi	7.7 ± 1.9	6.0 ± 1.5*
pH	7.04 ± 0.04	6.99 ± 0.05**
*After exercise:*		
PCr change	14.0 ± 3.3	13.7 ± 5.7
pH change	0.46 ± 0.29	0.18 ± 0.17*
*T*_“0.5 *PCr*”	59.6 ± 16.0	45.1 ± 11.0
*T*_“0.5 *Pi*”	34.8 ± 12.0^ +^	26.7 ± 7.0^ +^
*V*_“*max*”	0.25 ± 0.08	0.27 ± 0.19

*P < .05 and **P < .01 for arm versus leg. ^+^ *P* < .05 for *T*_0.5 PCr_ versus *T*_0.5 Pi_.

## Abbreviations

BC: Body Composition
BMD: Bone Mineral Density
DEXA: Dual-Energy X-ray Absorptiometry
GLP-2: Glucagon-like peptide 2
HPN: Home Parenteral Nutrition
SBS: Short Bowel Syndrome
NMR: Nuclear Magnetic Resonance
PTH: Parathyroid Hormone
